# Interactions Among Non-Coding RNAs in Diabetic Nephropathy

**DOI:** 10.3389/fphar.2020.00191

**Published:** 2020-03-03

**Authors:** Tamil Selvi Loganathan, Siti Aishah Sulaiman, Nor Azian Abdul Murad, Shamsul Azhar Shah, Abdul Halim Abdul Gafor, Rahman Jamal, Noraidatulakma Abdullah

**Affiliations:** ^1^ UKM Medical Molecular Biology Institute, Universiti Kebangsaan Malaysia, Kuala Lumpur, Malaysia; ^2^ Department of Community Health, UKM Medical Centre, Universiti Kebangsaan Malaysia, Kuala Lumpur, Malaysia; ^3^ Nephrology Unit, Faculty of Medicine, UKM Medical Centre, Universiti Kebangsaan Malaysia, Kuala Lumpur, Malaysia

**Keywords:** lncRNA, miRNA, circRNA, diabetic nephropathy, biomarkers, kidney disease

## Abstract

Diabetic Nephropathy (DN) is the most common cause of End-stage renal disease (ESRD). Although various treatments and diagnosis applications are available, DN remains a clinical and economic burden. Recent findings showed that noncoding RNAs (ncRNAs) play an important role in DN progression, potentially can be used as biomarkers and therapeutic targets. NcRNAs refers to the RNA species that do not encode for any protein, and the most known ncRNAs are the microRNAs (miRNAs), long noncoding RNAs (lncRNAs), and circular RNAs (circRNAs). Dysregulation of these ncRNAs was reported before in DN patients and animal models of DN. Importantly, there are some interactions between these ncRNAs to regulate the crucial steps in DN progression. Here, we aimed to discuss the reported ncRNAs in DN and their interactions with critical genes in DN progression. Elucidating these ncRNAs regulatory network will allow for a better understanding of the molecular mechanisms in DN and how they can act as new biomarkers for DN and also as the potential targets for treatment.

## Introduction

Diabetic nephropathy (DN) is the most common complication of both Type 1 and 2 diabetic patients and often results in End-stage renal disease (ESRD) (Gheith et al., 2016). Approximately about 20%–40% of diabetic patients will eventually develop DN and kidney disease, and this prevalence is depending on the population and ethnicity (Gheith et al., 2016). Despite advances in the diagnosis and treatment tools, DN remains a clinical burden. Early clinical diagnosis of DN is usually based on the presence of microalbuminuria (30–300 mg/day) or by the urinary albumin to creatinine ratio (> 30 mg/g of creatinine) (American Diabetes Association, 2019). However, in some patients with normoalbuminuria, a reduced glomerular filtration rate (GFR) was observed (American Diabetes Association, 2019), indicating that significant glomerular damage has already occurred before the albumin detection. Recent classification of DN stage progression showed that the clinical diagnosis of urinary albumin excretion (UAE) level is already in the third stage of the DN progression (Gheith et al., 2016). Therefore, identification and characterization of early biomarkers for DN become a priority. In this mini-review, we discussed the role of noncoding RNAs (ncRNAs) in DN progression and how they co-regulate each other and their target genes. Understanding of these ncRNAs regulatory networks in DN will allow for the discovery of new biomarkers and potential targets for treatment.

## Noncoding RNAs (ncRNAs)

In a single cell, the complex molecular characteristics are due to the transcriptomic profile that includes the protein-coding RNAs and the non-coding RNAs (ncRNAs) expression. Our understanding of the central dogma of the biology is that the information from the DNA is transcribed into the RNA and then are translated to proteins though, recent evidence shown that our genome consists of more ncRNAs than the protein-coding RNAs, and these ncRNAs are functional (Bertone et al., 2004; Djebali et al., 2012). These observations open up the new paradigm in understanding gene expression and regulation, particularly the multiple and diverse layers of molecular management of a single gene (Cipriano and Ballarino, 2018; Sulaiman et al., 2019). To date, various classes of the ncRNAs have been discovered. Most of these ncRNAs are generally grouped based on their sizes, which is the small ncRNAs (< 200 nucleotides) that include the microRNAs (miRNAs) and some of the circular RNAs (circRNAs) (Alvarez and DiStefano, 2013; Bhat et al., 2016; He et al., 2017), and the large ncRNAs such as the long ncRNAs (lncRNAs) (Bhat et al., 2016).

### MicroRNAs (miRNAs)

MiRNAs are the short single-stranded ncRNAs (size: 19–25 nucleotides), and their biogenesis and production have already extensively discussed before (Winter et al., 2009; Krol et al., 2010; Ha and Kim, 2014; O’Brien et al., 2018). The majority of the miRNAs are produced by RNA polymerases to generate the pri-miRNA transcripts from their genes in intronic clusters regions within the protein-coding genes (Rodriguez et al., 2004). Though some miRNAs do come from their genes, and some are within the lncRNAs (Rodriguez et al., 2004). Following that, the microprocessor complex (Drosha and DGCR8) cleaves the pri-miRNA to produce the distinctly hairpin structure of the precursor-miRNA (pre-miRNA) (Rodriguez et al., 2004). This pre-miRNA is processed further in the cytoplasm by RNase III endonuclease Dicer to remove the stem-loop structure to produce the mature miRNA duplex (Rodriguez et al., 2004). One of the mature miRNA duplex strands (5p or 3p strand) is incorporated into the functional miRNA-induced silencing complex (miRISC), which will bind to target messenger RNA (mRNAs) *via* complementary binding (Krol et al., 2010). A perfect complementary binding between the miRISC and the target mRNA will result in the mRNA degradation, whereas the incomplete complementary binding will lead to the translation repression (Rodriguez et al., 2004).

Studies have shown that miRNA-mediated gene regulation is a complex and dynamic process. A single miRNA could regulate many target genes at a time, and a single target mRNA could also be regulated by several miRNAs (Chowdhury et al., 2014; O’Brien et al., 2018), therefore implying the existence of the coordinated miRNA network in regulating the gene expression. Moreover, numerous evidence showing that the miRNA expression, regulatory action, and localization can change due to cellular environments (Ebert and Sharp, 2012; Kucherenko and Shcherbata, 2018). Serum starvation in multiple human cell lines has demonstrated that a significant decrease in intracellular miRNA levels with higher extracellular releases of these miRNAs either in vesicle form or as in circulating free miRNAs (Wang et al., 2010). Importantly, due to being released into extracellular fluids, these miRNAs may, therefore, can be used as biomarkers for disease.

### Long Noncoding RNA (lncRNAs)

Long noncoding RNAs (lncRNAs) refer to a group of large ncRNAs (size: > 200 nucleotides) that do not have any protein translation capacity (Quinn and Chang, 2015). Similar to mRNA production, lncRNAs are transcribed by the RNA polymerase II as the products of the alternative cleavage and splicing (Quinn and Chang, 2015; Yamamura et al., 2018). Though unlike mRNA, lncRNAs do not necessarily require polyadenylation for their functions, and in fact, some lncRNAs exist in both polyadenylated and non-polyadenylated forms (Derrien et al., 2012; Yamamura et al., 2017). The current classification of the lncRNAs is made based on their structure or size, localization, function, and interactions with other components (Dhanoa et al., 2018; Yao et al., 2019). Most of these lncRNAs come from the unconserved regions of the genome, such as the intergenic, exonic, or the distal protein-coding regions (Dhanoa et al., 2018; Yao et al., 2019). The secondary and tertiary structures of lncRNAs are quite conserved when compared to its primary structure (Quinn and Chang, 2015; Dhanoa et al., 2018). Due to their genomic origins, lncRNAs are, therefore, very difficult to characterize and highly diverse. In general, lncRNAs are grouped into five classes depending on their origins: 1) sense lncRNAs, 2) anti-sense lncRNAs, 3) bi-directional lncRNAs, 4) intronic lncRNAs, and 5) intergenic lncRNAs (Dhanoa et al., 2018).

In terms of their functions, lncRNAs can modulate gene expression at the transcriptional, post-transcriptional, and translational levels. Both cis- and trans-regulation of lncRNAs have been reported, in which the cis-regulatory lncRNAs exert their function on neighboring genes whereas the trans-regulatory lncRNAs exert their function to distant genes from their transcription sites (Yao et al., 2019). Generally, there are five modes of lncRNA functions. The first one is that lncRNA acts as the microRNA sponge or also known as the naturally competing endogenous RNAs (ceRNAs), in which the lncRNA can bind to a single microRNA with a complementary sequence and silences its-mediated actions towards the target mRNAs (Thomson and Dinger, 2016). This cross-talk and co-regulation of ceRNAs have garnered new interests to unravel the multi-layered molecular regulation of a common target mRNA. The second function is that lncRNA could act as a signal due to the environmental stimulus and initiates or suppresses the transcription process by interacting with transcription factors or chromatin-modifying enzymes (Dhanoa et al., 2018; Yao et al., 2019). The third function is that lncRNA can act the molecular decoy by interacting with the target protein and prevents its action (Dhanoa et al., 2018; Yao et al., 2019). Fourth, the lncRNA acts as a guide for the localization of specific proteins or transcription factors and therefore activates or deactivates the genes. Lastly, the fifth function is that lncRNA acts as a scaffold platform or adaptor to allow for two or more proteins or RNA molecules to interact with each other and initiate the downstream effects (Dhanoa et al., 2018; Yao et al., 2019).

The majority of the lncRNAs are localized in the nucleus, in which they are enriched at the chromatin region to recruit the chromatin-modifying enzymes (Deniz and Erman, 2017; Yao et al., 2019). In some lncRNAs, a presence of a short sequence from Alu elements allows for the interactions with a nuclear matrix protein, HNRNPK, and consequently causing these lncRNAs pre-dominantly to localize in the nucleus (Lubelsky and Ulitsky, 2018). Whereas, some lncRNAs move into the cytoplasm, in which they usually regulate the mRNA translation and post-transcription modifications (Yao et al., 2019). LncRNA functions as the ceRNAs or molecular decoys are the classic example of mRNA translation regulation in the cytoplasm. Evidence of extracellular lncRNAs are limited, but several studies have managed to show that lncRNAs, similar to miRNAs, exist in the vesicle-form or circulating free in biological fluids (Kelemen et al., 2019). Importantly, most lncRNAs express at low levels usually, thus at a specific stage of the disease progression, their expressions arise (Bhat et al., 2016; He et al., 2017).

### Circular RNAs (circRNAs)

A back-splicing event during the transcription process that ligates a splice donor site at the upstream to an acceptor sites at downstream of the RNA transcript, produces a covalently closed loop RNA product that does not contain either 5’ to 3’ polarity nor a poly-adenylated tail, and this is known as the circular RNA (circRNA) (Cao et al., 2018; Fang et al., 2018; Sulaiman et al., 2018; Hu et al., 2019). Due to its circular structure, circRNA is very stable and resistant to degradation by exoribonucleases (Liu et al., 2017; Cao et al., 2018). Similar to other ncRNAs, circRNAs do not encode for any protein products, and their biogenesis and production are well-regulated by cis-elements and trans-factors (Chen and Yang, 2015; Chen, 2016). Depending on their origins, the circRNAs are classified into four groups: 1) exonic circRNAs (ecircRNAs), 2) intronic circRNAs (ciRNAs), 3) exon-intron circRNAs (EIciRNAs), and 4) intergenic circRNAs (Memczak et al., 2013; Zhang et al., 2013; Li et al., 2015). As for their sizes, circRNAs sizes range from a few hundred to thousands of nucleotides (Chen and Yang, 2015).

CircRNAs are highly conserved across the eukaryotes (Conn et al., 2015), and like lncRNAs, their expressions are usually low but can be tissue- and developmental stage- or disease-specific (Abu and Jamal, 2016). Although circRNAs are known much earlier, its function and potential as biomarkers are not well understood. The most established function of circRNA is that circRNAs can act as a sponge to miRNAs to regulate gene expression (Cao et al., 2018; Fang et al., 2018; Hu et al., 2019), and in some cases, with proteins (Du et al., 2016). In terms of the circRNAs localization, some exonic circRNAs are abundantly present in the cytoplasm (Jeck et al., 2013), but no other information is available for other groups of circRNAs. However, many studies showed that circRNAs do present in extracellular vesicles and in circulating free form (Hon et al., 2019). Identification of the circRNAs’ role in the regulation of gene expression has expanded the understanding and knowledge about the complex interactions between these molecular regulators, which includes the miRNAs, lncRNAs, and circRNAs.

## Diabetic Nephropathy Progression

Progression to DN in diabetic patients is a very complicated process and is due to multiple factors, and previous reviews have discussed this topic extensively (Remuzzi et al., 2005; Piero et al., 2010; Zhang et al., 2017). As a summary, in diabetic settings, metabolic dysregulation leads to the alteration of kidney hemodynamics, promotes glomerulosclerosis, tubulointerstitial inflammation, and fibrosis. Both hyperglycemia and renal hypertension are the most known mediators for DN progression (Tziomalos and Athyros, 2015). Hyperglycemia activates the polyol pathway, hexosamine pathway and protein kinase C (PKC), which results in the accumulation of intracellular advanced glycation end products (AGEs), glomerular hyperfiltration, and hypertension (Stephen et al., 2004; Shaheer et al., 2017; Lin et al., 2018). A key hallmark event during this stage is the activation of the renin-angiotensin system (RAS) following the changes in GFR (Chawla et al., 2010; Ahmad et al., 2019). Elevated GFR is observed with glomerular and renal hypertrophy, and these events cause an increase in intraglomerular pressure (Chawla et al., 2010). Activation of RAS cascade (from pre-renin to Angiotensin-aldosterone) leads to glomerular afferent arteriole dilatation to increase renal blood flow as a feedback mechanism to maintain GFR. Though in diabetic individuals, hyperinsulinemia, and local Angiotensin II (AngII)-aldosterone also stimulates sodium reabsorption in the proximal tubule, mesangial cell constriction, and efferent arteriolar vasoconstriction (Chawla et al., 2010). Consequently, these events further increase intraglomerular pressure, tubulointerstitial inflammation, and worsen kidney damage (Chawla et al., 2010). A high level of AngII-aldosterone also stimulates reactive oxygen species (ROS) production, which further damage podocyte and tubular cells (Chawla et al., 2010). Increased glucose metabolism due to hyperglycemia also causes excessive production of ROS, which is highly potent and could damage the DNA and mitochondria (Stephen et al., 2004; Lin et al., 2018). The activation of the growth factors such as Transforming growth factor‐β (TGFB) and inflammatory cytokines *via* PKC, mitogen-activated protein kinase (MAPK), and transcription factor nuclear factor-κB (NF-κB) result in the downstream activation of tumor necrosis factor-α (TNFA) signaling and promotes cells repair and remodeling, which further aggravates the kidney lesion and fibrosis (Lin et al., 2018). Eventually, these uncontrolled and repeated kidney damages will lead to total kidney failure.

## Noncoding RNAs (ncRNAs) in DN

### Renal Hypertrophy and ECM Accumulation

The presence of TGFB in the kidney usually indicates a progression towards the renal hypertrophy and extracellular matrix (ECM) accumulation, together with evidence of RAS activation during renal hypertension Recent findings in DN patients and animal models have revealed that some of these ncRNAs regulate these early events in DN development (Bhat et al., 2016; Leti and DiStefano, 2017). Characterization of these ncRNAs in DN progression will allow for the discovery of early biomarkers for disease diagnosis and potential therapeutic targets, as these ncRNAs’ expressions may reflect the early disease development (Alvarez and DiStefano, 2013; Bhat et al., 2016; He et al., 2017).

#### MicroRNAs in Renal Hypertrophy and ECM Accumulation

MicroRNAs (miRNAs) are the most studied ncRNAs in renal-associated complications (Simpson et al., 2016) (**Table 1**). Recent systematic review and meta-analysis in 12 of previous DN human studies (Wang et al., 2019b), showed that 15 miRNAs were upregulated in DN, including miRNA-21, miRNA-181b, and miRNA-215. Whereas, seven miRNAs were downregulated in DN, including miRNA-26a, miRNA-126, miRNA-574-3p, miR-155, and miR-192 (Wang et al., 2019b). Importantly, the UAE rates correlated with these six miRNAs expressions (miR-133b, miR-342, miR-30, miR-192, miR-194, and miR-215), urinary albumin-creatinine ratio associated with five miRNAs expressions (miR-192, miR-217, miR-15b, miR-34a, and miR-636), and the GFR correlated with twelve miRNAs expressions (Eissa et al., 2016b; Wang et al., 2019b).

**Table 1:** List of the reported noncoding RNAs in Diabetic Nephropathy.

Noncoding RNA	Expression	Sample types	Origin/population	Molecular target	Function—pathogenesis
*MicroRNAs*					
miR-15b	Up	Urine exosome	T2DM patients—albuminuria and non-albuminuria (Eissa et al., 2016a)	NA	DN changes
		Podocyte cells	Mouse cell line (Fu et al., 2019)	*TNF α, IGFBP3, SEMA3A, IL1B, IL6*	
miR-20b	Up	Podocyte cells, HEK293T cells	Mouse and human cell line (Wang et al., 2017)	*SIRT7*	Podocyte cells apoptosis
miR-21	Up	renal cortical tissue	db/db DN mice (strain: C57BL/6JLepr) (Zhang et al., 2009)	*PTEN, AKT, PIK3CA*	Renal cell proliferation and hypertrophy
		Renal tissue, podocyte cells, HEK293T cells	DN patients, DN STZ Sprague-Dawley rats, mouse cell line, and human cell line (Chen et al., 2018)	*IL1B, TNF α, TIMP3*	Podocyte cells inflammatory and apoptosis
		Renal tissue, podocyte cells, HEK293T cells	C57BL/6J < sp > Lepr</sp> (Wang et al., 2019a)	*FOXO1, MAP1LC3A*, KHDRBS1, *P62*	Podocyte cells apoptosis and autophagy inhibition
miR-22	Up	Renal tissue, NRK-52E cells	STZ Sprague-Dawley rats (Zhang et al., 2018)	*PTEN, COL4, α-SMN1*	Inhibit autophagy process and renal fibrosis
miR-27a	Up	Kidney tubular epithelial cells, renal tissues	T2DM patients, rat cell line and diabetic Sprague Dawley rats (Hou et al., 2016)	*PPARG, Tgfb1, Smad3, Ccn2, Fn1, COL1*	Renal fibrosis
		NRK-52E, HK-2, HBZY-1 cell lines, renal tissues,	db/db diabetic and cell lines (Song et al., 2018)	*Gabpa, Itln1*	The pro-inflammatory response, oxidative stress
		podocyte cells	T2DM patients with DN, murine cell line and diabetic rats (Zhou et al., 2017)	*Pparg, Ctnnbip1, Snai1, Acta2*	Podocyte cells injury and apoptosis
miR-34a	Up	Urine exosomes	T2DM patients—albuminuria and non-albuminuria (Eissa et al., 2016a)	NA	DN changes
miR-130a	Up	Urine exosomes, renal tissues, human mesangial cells	T1DM patients with DN, DN mice, and human cell line (Barutta et al., 2013)	NA	DN changes
miR-130b	Up	Renal tissues, mesangial cells	DN patients and cell line (Ma et al., 2019)	*TGFB1, COL1, COLIV, FN, t‐Smad2/3, p‐Smad2/3, and SMAD4*	Renal fibrosis, and extracellular matrix accumulation
miR-133b	Up	Urine exosomes	T2DM patients—albuminuria and non-albuminuria (Eissa et al., 2016b)	*TGFB1*	DN changes
		Renal cortex, human proximal tubule cell line, HEK293T cells	OLETF mice and human cell line (Sun et al., 2018c)	*CDH1, TGFB1, COL1, FN, ACTA2, SIRT1*	Epithelial-mesenchymal transition, and renal fibrosis
miR-134-5p	Up	Renal tissues, podocyte cells	Db/db mice (Qian et al., 2018)	*BCL2*	Podocytes apoptosis
miR-135a	Up	Serum, renal tissues, mesangial cells, HEK293T cells	DN patients, healthy control, and db/db mice, cell line (He et al., 2014)	*TRPC1, FN, COL1, TGFB1, PCNA, CCND1, P21, CDH, VIM*	Mesangial cells proliferation, increase extracellular matrix proteins, and renal fibrosis
miR-141	Up	Mesangial cells	Human cell line (Li et al., 2018b)	*IRS2*	Renal hypertrophy, inflammation, and cell apoptosis
miR-145	Up	Urine exosomes, renal tissues, human mesangial cells	T1DM patients with DN, DN mice, and human cell line, (Barutta et al., 2013)	*STAT1, TGFB1*	Mesangial cells damage
miR-155-5p	Up	Proximal tubular cells	Human Cell line (Wang et al., 2018e)	*SIRT1, LC3 II, ATG5*	Autophagy
miR-181a	Up	Plasma	DN patients (Bijkerk et al., 2015)	NA	Microvascular damage
miR-181b	Up	Glomerular mesangial cells, renal biopsy	DN patients (Zhu et al., 2019)	*TIMP3*	Mesangial cells apoptosis
miR-182-5p	Up	Renal biopsy, podocyte cells	DN patients and cell line (Ming et al., 2019)	*CD2AP, FOXO1, BCL2*	Podocyte cells injury and apoptosis
miR-192	Up	Renal cortical tissues, mesangial cells	db/db mice (Kato et al., 2009)	*Tgfb1, Akt, Pten, E box*	Glomerular hypertrophy, mesangial cells expansion, Renal fibrosis, extracellular matrix accumulation, and tubular damage Glomerular hypertrophy and mesangial cells expansion
		Urine extracellular vesicles, renal tubular epithelial cells, mesangial cells,	Albuminuria patients and human cell line (Jia et al., 2016)	*TGFB1*	Tubular and mesangial cells damage
		Renal biopsy	DN patients (Krupa et al., 2010)	*TGFB1, ZEB1, CDH1*	Tubulointerstitial fibrosis
miR-194	Up	Urine extracellular vesicles, human renal tubular epithelial cells, human mesangial cells	DN patients with T2DM and human cell line (Jia et al., 2016)	*AKT, TRAF6*	Tubular and mesangial cells damage
miR-195	Up	Plasma and urine	CKD patients (Sayilar et al., 2016)	NA	DN changes
miR-199b	Up	Renal cortex, human proximal tubular epithelial cells	OLETF mice and human cell line (Sun et al., 2018a)	*CDH1, TGFB1, COL1, FN, ACTA2, SIRT1*	Epithelial-mesenchymal transition, and renal fibrosis
miR-200	Up	Renal cortex tissues, mesangial cells	db/db mice (Park et al., 2013)	Fog2*, Pi3k, Tgfb1*	Glomerular mesangial hypertrophy
miR-212	Up	Renal, plasma	Sprague-Dawley rats (Eskildsen et al., 2013)	*Angiotensin, MAPK3*	Hypertension in renal
miR-215	Up	Urine extracellular vesicles, renal tubular epithelial cells, mesangial cells	Albuminuria patients and human cell line (Jia et al., 2016)	*TGFB1*	Tubular and mesangial cells damage
miR-216a	Up	Renal cortex tissues, mesangial cells	db/db mice (Kato et al., 2009)	*Tgfb, Akt, Pten, Zeb1*	Glomerular hypertrophy and mesangial cells expansion
miR-217	Up	Renal cortex tissues, mesangial cells	db/db mice (Kato et al., 2009)	*Tgfb, Akt, Pten, Zeb1*	Glomerular hypertrophy and mesangial cells expansion
miR-320	Up	Renal cortex tissues, podocyte cells	Db/db mice (He et al., 2019)	*Mafb*	Podocyte cells injury and apoptosis
miR-324-3p	Up	Glomerular cells, tubular cells	Munich Wistar Fromter mice (Macconi et al., 2012)	Angiotensin, Ac-SDKP*, Prep*	Renal fibrosis
miR-337	Up	Renal tissues, mesangial cells	DN mice and human cell line (Wang et al., 2008)	*TGFB*	Mesangial cells expansion, and renal toxicity and fibrosis
miR-342	Up	Urine exosomes	T2DM patients—albuminuria and non-albuminuria (Eissa et al., 2016b)	*TGFB1*	DN changes
miR-370	Up	Renal tissues, mesangial cells	DN mice (Yu et al., 2019)	*Cnpy1*	Mesangial cells proliferation and extracellular matrix accumulation
miR-377	Up	Renal tissues, mesangial cells	DN mice and human cell line (Wang et al., 2008)	*FN, PAK1, SOD1, SOD2, TGFB*	Renal toxicity and fibrosis
		Renal cortex, mesangial cells	T2DM db/db mice and mice cell line (Kato et al., 2016)	*Tgfb1, Akt, Erk, Chop, Edem3*	Endoplasmic reticulum stress, and glomerular extracellular matrix and hypertrophy
miR-379	Up	Renal tissues, mesangial cells	db/db mice and mouse cell line (Kato et al., 2016)	*Tgfb1, Akt, Erk, Chop, Edem3*	Renal toxicity and fibrosis, endoplasmic reticulum stress, extracellular matrix accumulation, and podocyte cells injury
miR-382	Up	Renal tissue, mesangial cells	STZ mice (Wang et al., 2018c)	*Foxo1*	Renal hypertrophy and extracellular matrix accumulation
miR-4449	Up	Serum exosomes	DN patients (Kim et al., 2019)	NA	Angiogenesis
miR-451	Up	Plasma and urine	CKD patients (Sayilar et al., 2016)	NA	DN changes
		Renal tissues, primary mouse mesangial cells	DN mice, and cell line (Zhang et al., 2012),	Ywhab, P38 Mapk	Renal fibrosis and glomerular cells proliferation
miR-494	Up	Renal tissues, mesangial cells	db/db mice and cell line (Kato et al., 2016)	*Tgfb1, Akt, Erk, Chop*	Renal toxicity and fibrosis
miR-495	Up	Renal tissues, mesangial cells	db/db mice and cell line (Kato et al., 2016)	*Tgfb1, Akt, Erk, Chop*	Renal toxicity and fibrosis
		Urine	T1DM patients with DN (Christos et al., 2015)	NA	DN changes
miR-503	Up	Renal tissues, podocyte cells	STZ mice (Zha et al., 2019)	*E2f3*	Podocyte cells injury and apoptosis
miR-636	Up	Urine exosome	T2DM patients—albuminuria and non-albuminuria (Eissa et al., 2016a)	NA	DN changes
miR-770-5p	Up	Podocyte cells	Mice cell line (Zhang et al., 2019)	*Triap1*	Podocyte cells apoptosis and cells death
miR-181a	Down	Renal tissue	Hypertensive and normotensive patients (Marques et al., 2011)	*AIFM1, APOE, RENIN*	Hypertension in renal
miR-25	Down	Serum, renal tissue, and HK-2 cells	DN patients and human cell line (Li et al., 2017a)	*PTEN, AKT*	Renal tubular epithelial cell injury and apoptosis, mesangial cells injury, and glomerular hypertrophy
		Renal tissue	STZ Sprague-Dawley rats (Fu et al., 2010)	*NOX4*	
		Glomeruli, mesangial cell	STZ mice and cell line (Oh et al., 2016)	*p-MeCP2, Hipk2, Tgfb*	Oxidative stress
miR-26a	Down	Renal biopsy, STZ mice, and human podocyte cells	DN patients, STZ mice, and human cell (Koga et al., 2015)	*TGFB, CTGF, SMAD, CTDSP2, CTDSPL*	Extracellular matrix accumulation
miR-29a	Down	Glomeruli, podocyte cells	STZ mice (Lin et al., 2014)	Nephrin	Podocyte cell apoptosis
		Mesangial cells	Mice and cell line (Tung et al., 2019)	*Cb1r, Pparg*	Renal hypertrophy
miR-30	Down	Urine exosomes	T2DM patients—albuminuria and non-albuminuria (Eissa et al., 2016b)	NA	DN changes
miR-93	Down	Renal microvascular endothelial cells, podocyte cells	Mice cell line and diabetes db/db mice (Long et al., 2010)	*Vegfa, Mcm7*	Podocyte cells damage
		Podocyte cells, renal tissues	Mice cell line and Cg-Dock7^m+/+^Lepr^db/J^ mice (Badal et al., 2016)	*Msk2*	
		Renal tissue, human renal tubular cells	DN patients and human cell line (Ma et al., 2018)	*ORAI1, TGFB1*	Renal fibrosis and endothelial mesenchymal transition
miR-155	Down	Urine exosomes, blood, renal tissues	DN patients and C57BL6/J mice (Barutta et al., 2013)	NA	Podocyte cells injury
miR-205	Down	Renal tubules epithelial cells	Human cell line (Muratsu-Ikeda et al., 2012)	*EGLN2, VEGF GLUT1, HIF-1α*	Renal injury
miR-214	Down	Renal, human renal proximal tubular epithelial cells	DN mice and human cell line (Yang et al., 2019)	*UCP2*	Oxidative stress
miR-374a	Down	Renal biopsy, human proximal tubular epithelial cells	DN patients and human cell line (Yang et al., 2018b)	*CCL2*	Induce inflammatory response
miR-423-5p	Down	Renal biopsy, podocyte cell	DN patients and mice cell line (Xu et al., 2018)	*NOX4*	Oxidative stress and podocyte cells death
miR-424	Up	Urine exosomes, renal tissues, human mesangial cells	T1DM patients with DN, DN mice, and human cell line, (Barutta et al., 2013)	NA	DN changes
miR-455-3p	Down	Mesangial cells, proximal tubular epithelial cells	Human cell line (Wu et al., 2018)	*ROCK2*	Inflammation and fibrosis
miR-574-3p	Down	Plasma	DN patients (Bijkerk et al., 2015)	*ANGPT2/ANGPT1, IGF1*	Effects renal function (eGFR and creatinine)
miR-663	Down	Renal tissue	Hypertensive and normotensive patients (Marques et al., 2011)	*AIFM1, APOE, RENIN*	Hypertension in renal
miR-874	Down	Renal cortex	STZ mice (Yao et al., 2018)	*TLR4, IL6, IL1B,* *TNFA*	Podocyte cells proliferation and apoptosis
Let-7a-5p	Down	Renal tissue, mesangial cells	db/db mice and cell line (Wang et al., 2019c)	*HMGA2*	Renal hypertrophy and apoptosis
*Long noncoding RNAs*					
ERBB4-IR	Up	Renal tissue	C57BL/6J mice (Zhou et al., 2014)	*Smad3, Tgfb*	Renal fibrosis
		Renal cortex, mesangial cells, tubular epithelial cells	db/db mice and mice cell (Sun et al., 2018b) (Sun et al., 2018b)	*Smad3, Tgfb1, miR-29b*	
GAS5	Up	Human renal tubular epithelial cell	Human cell line (Lv et al., 2019)	*miR-27a, P53, CASP3, BNIP3*	Renal tubular epithelial cells apoptosis
Gm4419	Up	High glucose mesangial cells	Mice cells (Yi et al., 2017)	*Nfkb*	Renal inflammation and fibrosis.
GM5524	Up	Renal tissues, podocyte cells	db/db mice (Feng et al., 2018)	*CASP3, BAX,* LC3I, LC3II *Atg5, Atg7, Bcl2*	Podocyte cells apoptosis and cells autophagy
GM6135	Up	Mesangial cells	db/db mice (Ji et al., 2019)	*TLR4, miR‐203*	Mesangial cells proliferation and apoptosis
LncRNA CJ241444-miR-192	Up	Renal cortical tissues, mesangial cells	Obese type 2 diabetic db/db mice and mouse cells (Kato et al., 2013)	*Tgfb, Akt, Col1a2, Col4A1, Smad, Ets1, miR-192*	Mesangial cells fibrosis
Lnc-MGC	Up	Renal tissue, mesangial cells	STZ mice and human cell (Kato et al., 2016)	*TGFB1, SMAD, CHOP*	Renal toxicity and fibrosis, glomerular hypertrophy, extracellular matrix accumulation, and podocyte cells injury
LncRNA NR_033515	Up	Serum, HEK293 T cells,mesangial cells	DN patients and mouse cell (Gao et al., 2018)	*HAVCR1, PCNA, CCND1TGFB1, P38, ASK1, FN, ACTA2, CDH, VIM, miR-743b-5p*	EMT and cell proliferation
Metastasis associated lung adenocarcinoma transcript 1 (MALAT-1)	Up	Umbilical vein endothelial cells, STZ mice	Human cell line and mice (Puthanveetil et al., 2015)	*SAA3, TNF-α, IL-6*	Renal inflammation and fibrosis Microvascular inflammation
		Podocyte cells, HEK‐293 cells	Human, C57BL/6 mice, cell line (Hu et al., 2017)	*CTNNBIP1, SRSF1*	Podocyte cells damage
		Renal tissues, proximal tubular epithelial cells	Sprague Dawley rats and human cell line (Li et al., 2017b)	*miR-23c, ELAVL1, NLRP3*	Renal tubular epithelial cell pyroptosis
		Serum	ESRD patients with diabetes (Fawzy et al., 2018)	*miR-499a*	DN changes
NEAT1	Up	Renal tissues	Diabetic mice (Huang et al., 2019)	*Akt, Mtor, Tgfb1, Fn, ColIv*	Mesangial cell proliferation and fibrosis
		Renal tissues	Sprague–Dawley rats (Wang et al., 2018d)	*Zeb1, miR‐27b‐3p, Ask1, fibronectin, Tgfb1*	
Plasmacytoma variant translocation (PVT1)	Up	Blood	Gila River Indian Community with T2DM and DN T1DM patients with DN (Hanson et al., 2007)	NA	DN changes
		Blood, human primary renal cell	Pima Indians with T1DM and T2DM, and human cell line	NA	
		Mesangial cells	Human cell line (Alvarez and DiStefano, 2011)	*FN1, COL4A1, SERPINE1, TGFB1*	Extracellular matrix accumulation and mesangial cells fibrosis
CASC2	Down	Serum, renal tissue	DN patients (Wang et al., 2018a)	NA	Podocyte cells deaths
		Serum, podocyte cells	DN patients and mouse (Yang et al., 2018a)	*JNK1*	
CYP4B1-PS1-001	Down	Mesangial cells, renal cortex	db/db mice (Wang et al., 2018b)	*Ncl*	Renal proliferation and fibrosis
		renal tissue, mesangial cells, HEK293T	C57BL/KsJ db/db mice, and human and mouse cell line (Wang et al., 2016a)	*PCNA, CCND1, FN, COLI*	Mesangial cells proliferation and fibrosis
ENSMUST00000147869	Down	Renal cortex	db/db and db/m mice (Wang et al., 2016b)	*Cyp4a12a*	Mesangial cells proliferation and fibrosis
GM15645	Down	Renal tissue, podocyte cells	db/db mice (Feng et al., 2018)	*CASP3, BAX,* LC3I, LC3II *Atg5, Atg7, Bcl2*	Podocyte cells apoptosis and cells autophagy
LINC01619	Down	Renal biopsy, cortex, podocyte cell	T2DM patients, and STZ Sprague Dawley rats (Bai et al., 2018)	*FOXO1, ROS, CHOP, GRP78, miR-27a*	Podocyte cells injury and apoptosis and endoplasmic reticulum stress
LncRNA 1700020I14Rik	Down	Renal tissue, mesangial cells	db/db and db/m mice (Li et al., 2018a)	*Sirt1, Hif1a, Col4, Fn, Tgfb1, miR-34a-5p*	Mesangial cells proliferation and fibrosis
lncRNA RIAN	Down	NIH3T3 cells and renal tissue	Mice and cell line (Bijkerk et al., 2019)	*Acta2, Col1a1, Smad2, Smad3, miR-150*	Myofibroblast formation
Taurine up-regulated 1 (TUG-1)	Down	Renal cortex, mesangial, podocytes cells	db/db and db/m mice and cell line (Long et al., 2016)	*Ppargc1a*	Podocyte cells death and extracellular matrix accumulation Reduce glomerular filtration rates and podocyte cells apoptosis
		Renal cortex, mesangial cells	C57BL/KsJ-db/db mice and mice cell line (Duan et al., 2017)	*miR-377, Pparg,* Serpine1*, Tgfb1, Fn, Col Iv*	Extracellular matrix accumulation of mesangial cells
		Renal tissues, podocyte cells	Sprague Dawley rats and mice cell line (Lei et al., 2018)	*Traf5*	Podocyte cells apoptosis
		Renal biopsy, podocyte cells	DN patients and human cell line (Shen et al., 2019)	PGC‐1α, CHOP	Podocyte cells apoptosis and endoplasmic reticulum stress
Myocardial infarction associated transcript (MIAT)	Down	Proximal convoluted tubule, proximal tubular epithelial cell line (HK-2)	STZ Wistar rats and cell line (Zhou et al., 2015)	*Nrf2, Acta2, Smad2/3, Col1a1*	Renal cell apoptosis and fibrosis
*Circular noncoding RNAs*					
circRNA_15698	Up	Renal cortex, mesangial cells	C57BL/KsJ‐db/db mice (Hu et al., 2019)	*Tgfb1, Col1a1*, *Col4a1* and *Fn, miR‐185*	Renal fibrosis

Among these reported miRNAs, some are shown to regulate RAS and renal hypertension (Hagiwara et al., 2013) (**Table 1**). For example, investigation in the human kidney cell line showed that miR-663 directly regulates the expression of renin (*REN*) and apolipoprotein E (*APOE*) genes, and miR-181a directly regulates the expression of *REN* and apoptosis-inducing factor, mitochondrion-associated-1 (*AIFM1*) genes (Marques et al., 2011). Inhibition of these miRNAs expression will increase their target genes expressions like the renin and, thus, induce the RAS cascade-production of angiotensin I/II and aldosterone, which is a significant dysregulation event in the early DN progression (Marques et al., 2011). Therefore, the reduction of both miRNAs’ expression (miR-181a and miR-663) in the patients can be used as the early biomarkers of DN, since their reduced expression may reflect the start of the RAS activation. Moreover, both miR-181a and miR-663 expressions were reduced in hypertensive renal tissue (Marques et al., 2011), and serum expression of miR-181a can be a biomarker for the renal miR-181a (Marques et al., 2015) thus, confirming that these miRNAs can serve as biomarkers for early dysregulation of RAS in DN patients. Besides that, Ang II itself can increase five downstream miRNAs expressions, including miR-29b, miR-129-3p, miR-132, miR-132-5p, and miR-212, as reported in a human kidney cell line study with the overexpression of Angiotensin II receptor type 1 (*AT1R*) (Jeppesen et al., 2011). Though, only some of these miRNAs are involved in DN, particularly the miR-29b and its role in kidney fibrosis and cell death (Sun et al., 2018a). Understanding the roles of miRNAs in RAS activation is important, as RAS blocker therapy or treatment is currently one of the main treatments for DN patients (Jeppesen et al., 2011).

Another early dysregulated miRNAs in DN progression is the miR-192 (Kato et al., 2009; Krupa et al., 2010; Jia et al., 2016; Andrea et al., 2018). MiR-192 is the most abundant miRNA in the kidney, in which its high expression protects the kidney function (Krupa et al., 2010). In DN patients and mice model, miR-192 expression was reduced as the DN progresses (Kato et al., 2009; Krupa et al., 2010; Jia et al., 2016). This reduction of miR-192 expression removed the suppression of the E box repressors, *Zeb1*, and *Zeb2* expressions, which are the inducers of the TGFB pathway, and further promoted TGFB-induced downstream activation of other miRNAs to promote renal hypertrophy and ECM accumulation (Kato et al., 2007). One example of the TGFB-downstream miRNAs is the miR-21, which is the most well-known miRNA in DN and its expression was upregulated in both human DN and mice models (Zhang et al., 2009; Sayilar et al., 2016; Chen et al., 2018; Wang et al., 2019a). MiR-21 negatively regulates the expression of Phosphatase and tensin homolog (*PTEN*) and matrix metalloproteinases (Chau et al., 2012). Hyperglycemia condition upregulates miR-21 expression in the plasma and urine samples (Sayilar et al., 2016). In a study of DN patients, miR-21 expression was elevated in the kidney biopsy and correlated with the podocyte cell damage, together with a reduced metalloproteinase inhibitor-3 (*TIMP3*) expression (Chen et al., 2018). *TIMP3* is an important regulator of a healthy kidney, and the reduction of its expression increases the podocyte cell death (Chen et al., 2018). Another target of miR-21 is the forkhead box O1 (*Foxo1*), which was down-regulated in DN-induced by hyperglycemia condition (Wang et al., 2019a). Treatment of a currently clinical-trialed DN drug, Atrasentan (an endothelin-1 receptor antagonist) reduced miR-21 expression, restored the *Foxo1* expression, and thus promoted renal autophagy (Wang et al., 2019a). Therefore, following TGFB response, majority of these downstream network of miRNA activates the PI3K/AKT/mTOR pathway, by targeting the *PTEN* (miR-216a, miR-217, miR-21, miR-22, miR-25, and miR-214) (Kato et al., 2009; Denby et al., 2011; Li et al., 2017a; Zhang et al., 2018), collagen type I alpha1 (*COLIA1*), Collagen IV (*COL4*), and Fibronectin (*FN*) (miR-130b) (Ma et al., 2019), PI3K inhibitor (*Fog2*) (miR-200 family) (Park et al., 2013), Tyrosine 3-monooxygenase (*Ywhab*) (miR-451) (Zhang et al., 2012), DEP domain-containing mTOR-interacting protein (*Deptor*) (miR-181a) (Maity et al., 2018), Protein canopy homolog-1 (*Cnpy1*) (miR-370) (Yu et al., 2019), high−mobility group AT−hook-2, *Hmga2* (let-7a-5p) (Wang et al., 2019c), Insulin receptor substrate-2, *IRS2* (miR-141) (Li et al., 2018b), forkhead box proteins, and *Foxo1* (miR-382) (Wang et al., 2018c) genes. Some of these miRNAs are found in circulating bio-fluids and therefore, could potentially be as biomarkers.

In contrast to the pro-DN miRNAs, miR-93 is the protective miRNA, with its expression was reduced in an animal model of DN, and miR-93 is a direct regulator of the vascular endothelial growth factor A (*Vegfa*) (Long et al., 2010) and *Msk2*, a ribosomal S6 kinase of serine/threonine (Badal et al., 2016) expressions. Overexpression of miR-93 prevented the chromatin remodeling in podocyte cells after induction of hyperglycemia condition, *via* the suppression of *Msk2* expression (a kinase that phosphorylates histone, H3S10) (Badal et al., 2016). Thus, making miR-93 as the potential therapeutic option to alleviate DN. Importantly, miR-93 also negatively regulates the Calcium release-activated calcium channel protein-1 (*ORAI1*), and reduction of miR-93 expression in DN caused an increase in *ORAI1* expression and subsequently promoted TGFB-mediated ECM accumulation and fibrosis (Ma et al., 2018). Excessive ECM accumulation will eventually lead to glomerulosclerosis; therefore, it is the first and crucial step for progressive renal function loss.

#### Long Noncoding RNAs in Renal Hypertrophy and ECM Accumulation

Several lncRNAs are involved in the regulation of renal hypertrophy and ECM accumulation, though the evidence is quite limited (**Table 1**). One such lncRNA is the Plasmacytoma Variant Translocation-1 (PVT1), which is the first lncRNA to be associated with DN patients (Millis et al., 2007; Alvarez and DiStefano, 2011). Hyperglycemia induced higher PVT-1 expression and caused mesangial cell expansion through ECM accumulation of the FN, TGFB1, type IV collagen (COL4A1), and type 1 plasminogen activator inhibitor (PAI1) (Alvarez and DiStefano, 2011), as well as the myosin (MYC) proteins (Hanson et al., 2007). The knockdown of PVT-1 expression significantly caused the reduction of these molecules (Alvarez and DiStefano, 2011). Another reported lncRNA is the Nuclear Enriched Abundant Transcript-1 (NEAT1), which is also highly expressed due to hyperglycemia, and NEAT1 interacts with AKT/mTOR pathway (Huang et al., 2019). Inhibition of NEAT1 expression in an animal model of DN causes a reduction of TGFB1, FN, and COL4A1 production (Huang et al., 2019). Similarly, lncRNA ERBB4-IR also promotes renal fibrosis *via* the activation of the TGFB/SMAD3 pathway (Zhou et al., 2014; Sun et al., 2018b). Interestingly, knockdown of this lncRNA ERBB4-IR in DN mice protected the mice from having high albuminuria and creatinine, as well as prevented renal fibrosis (Sun et al., 2018b).

Opposite to PVT-1 and NEAT-1, a hyperglycemic condition caused downregulation of lncRNA, CYP4B1-PS1-001 expression, which increases nucleolin (NCL) protein expression (Wang et al., 2016a; Wang et al., 2018b). An increase in this protective lncRNA expression reduces the production of FN, COL4A1, and proliferation markers (PCNA and CCND1) in DN mice (Wang et al., 2018b). Another protective lncRNA is the lncRNA ENSMUST00000147869 that negatively regulate ECM protein accumulation in a hyperglycemic condition in DN mice (Wang et al., 2016b). Though, the exact role of this lncRNA is unknown.

#### LncRNA-miRNA Interactions in Renal Hypertrophy and ECM Accumulation

**Figure 1:**
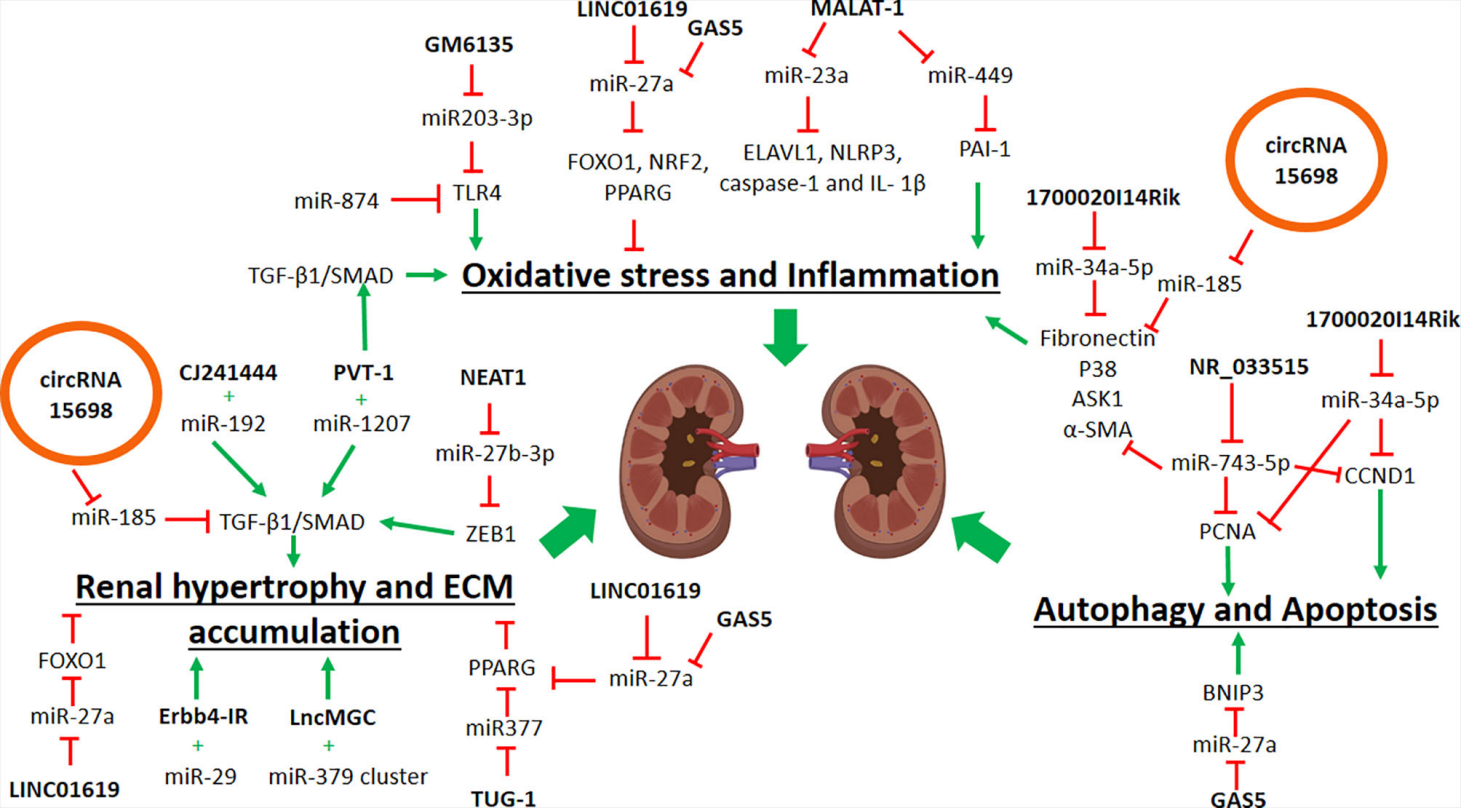
Schematic diagram showing the roles of noncoding RNAs in contributing to diabetic nephropathy. ECM, extracellular matrix; ER, Endoplasmic reticulum; black arrow, positive regulation; red arrow, negative regulation.

Understanding the interactions between miRNAs and lncRNAs to regulate genes involved in DN are important, as they will unravel the critical steps in DN progression. For example, in ECM accumulation (**Figure 1**), previous studies in DN mice showed that interactions of lncRNA CJ241444-miR-192 activates TGFB1/SMAD signaling (Kato et al., 2013) and lncRNA Erbb4-IR-miR-29b promotes the collagen genes expression, ECM accumulation and kidney injury (Sun et al., 2018b). In both of the studies (Kato et al., 2013; Sun et al., 2018a), the lncRNAs act as the miRNA sponge to exert the outcomes. Similarly, another reported lncRNA-miRNA interaction in DN is the lncRNA PVT-1 that mediates the ECM proteins accumulation *via* the actions of its-derived miRNAs, miR-1207-5p, and miR-1207-3p (Alvarez and DiStefano, 2011). Under the hyperglycemic condition, higher expression of both PVT-1 and its miRNAs led to an increase of the TGFB1/SMAD signaling pathway and further promoted the ECM protein accumulation and oxidative stress (Alvarez and DiStefano, 2011). Another example is the miR-379 cluster that consists of 40 miRNAs that are regulated by ER stress in DN, and lncRNA lncMGC is also hosted in this same cluster (Kato et al., 2016). LncMGC controls the expression of the miR-379 cluster, and the upregulation of this miRNAs cluster induces ECM accumulation and hypertrophy (Kato et al., 2016). Thus, inhibition of lncMGC expression could be used as a potential therapy for DN to reduce the miR-379 cluster’s effects, following the ER stress. Besides that, lncRNA NEAT1 inhibition is also a potential therapeutic therapy, as this NEAT1 inhibition leads to the suppression of ECM accumulation, *via* the reduction of ASK1, FN, and TGFB1 production (Wang et al., 2018d). This NEAT1-mediated ECM suppression was due to its interaction with miR-27b-3p, and its target, the TGFB inducer, *Zeb1*, in which NEAT1 is no longer suppressing the miR-27b-3p, which in turns activates the suppression of *Zeb1*, and thus can prevent DN progression (Wang et al., 2018d). Administration of the anti-apoptotic lncRNA, TUG-1, suppresses the miR-377 expression to increase the expression of miR-377’s target gene *PPARG* and thus prevents ECM accumulation in DN mice (Duan et al., 2017). Therefore, treatment to increase TUG-1 expression may be beneficial to prevent DN development, though further studies are needed to confirm this potential treatment. By understanding these interactions between lncRNAs and their target miRNAs, these findings will allow for therapeutic targets selection to prevent ECM accumulation and possibly DN progression.

### Oxidative Stress and Inflammation

#### MiRNAs in Oxidative Stress and Inflammation

As for inducing the oxidative stress, miR-205 directly regulates the expression of Egl-9 Family Hypoxia Inducible Factor 2 (*EGLN2*), and miR-205 expression was reduced due to oxidative stress (Muratsu-Ikeda et al., 2012). Upon reduction of miR-205 expression, *EGLN2* expression was increased and caused the reduction of the HIF-2A protein, and consequently increased the reactive oxygen species (ROS) level (Muratsu-Ikeda et al., 2012). Another oxidative stress-related miRNA is the miR-377, and its expression was higher in the renal tissue of DN mice (Wang et al., 2008), thus caused a reduction in the expression of superoxide dismutase 2 (*SOD2*), an enzyme responsible for removing free superoxide radicals. Several other miRNAs also regulate endoplasmic reticulum (ER) and oxidative stress in renal cells, such as miR-379 cluster, miR-494 (Kato et al., 2016), miR-15b (Eissa et al., 2016a; Fu et al., 2019; Wang et al., 2019b), and miR-135a (He et al., 2014). Interestingly, miR-214 expression was reduced in peripheral blood of DN mice and overexpression of miR-214 in human renal proximal tubular epithelial cells prevented oxidative stress *via* upregulation of mitochondrial uncoupling protein-2 (*UCP2*) expression and its downstream pathway, the ROS/AKT/mTOR signaling pathway with significant reduction of ROS (Yang et al., 2019). Since the miR-214 reduction in circulating blood samples of DN mice was consistent with the renal expression of miR-214, and its regulation of the oxidative stress in kidney cells, thus miR-214 can be used as a potential oxidative stress biomarker. Another miRNA is the miR-25 that negatively regulates NADPH oxidase-4 (*Nox4*) expression (an enzyme that produces ROS), and its expression was reduced in the renal tissue of DN rats (Fu et al., 2010). Importantly, the miR-25 expression is also regulated by Homeodomain-interacting protein kinase-2 (Hipk2) (Oh et al., 2016), indicating a complex regulation of HIPK2-miR-25-*NOX4* in the maintenance of ROS. Furthermore, *NOX4* gene expression is also regulated by miR-423-5p, that were downregulated in kidney tissues of DN patients (Xu et al., 2018), further re-affirm the complex and tight regulation of *NOX4* gene.

In terms of inflammation, miR-27a is a pro-inflammatory miRNA that negatively regulates the expression of nuclear factor erythroid 2-like 2 (*NRF2*) and peroxisome proliferator-activated receptor γ (*PPARG*), thus promotes the secretion of pro-inflammatory cytokines in DN animal and cell line models (Hou et al., 2016; Song et al., 2018; Zhou et al., 2018). Intriguingly, miR-27a expression is also negatively regulated by *Itln1*, an adipokine that usually reduced in metabolically dysregulated individuals (Song et al., 2018), indicating its role in metabolic syndrome. Another inflammatory miRNA is the miR-324-3p, and its expression was higher in glomerular and tubular tissues of DN mice (Macconi et al., 2012). Increased of this miR-324-3p expression led to renal inflammation *via* the suppression of prolyl endopeptidase gene expression (*Prep*), an enzyme that is responsible for regulating angiotensin metabolism (Macconi et al., 2012). In contrast to that, miR-374a is the anti-inflammatory miRNA in DN, which negatively regulates the expression of Monocyte chemo-attractant protein-1 (*CCL2*), a pro-inflammatory chemokine (Yang et al., 2018b). In DN patients, restoration of miR-374a expression in renal tubular epithelial cells prevented the inflammatory response (Yang et al., 2018b). Similarly, miR-874 negatively regulates the expression of Toll-like receptor-4 (*Tlr4*), and thus prevents the secretion of pro-inflammatory cytokines in DN mice (Yao et al., 2018). Another anti-inflammatory miRNA is the miR-455-3p that negatively regulates the expression of coil-containing protein kinase 2 (*ROCK2*), and consequently reduced the inflammatory cytokines and fibrosis markers in a human kidney cell line (Wu et al., 2018). Identification of these anti-inflammatory and oxidative stress inhibitor miRNAs will allow for the possible use of these miRNAs mimics to prevent or reduce inflammation and oxidative stress. Since prolonged inflammation will eventually lead to fibrosis and renal damage, early intervention is necessary to prevent more severe complications.

#### LncRNAs in Oxidative Stress and Inflammation

A few lncRNAs also regulate renal inflammation and oxidative stress, though limited evidence is available. LncRNA GM4419 is a regulator for NF-κB signaling by directly interacts with the p50 protein, and thus, an increase of its expression promotes the inflammatory responses in renal mesangial cells upon stimulation of high glucose (Yi et al., 2017). Inhibition of this lncRNA significantly led to the reduction of inflammation and fibrosis (Yi et al., 2017). Another reported pro-inflammatory lncRNA is the Metastasis-associated lung adenocarcinoma transcript-1 (MALAT-1) that was increased in DN mice and interacted with serum amyloid A3 (*Saa3*), subsequently promotes the production of inflammatory factors (Il6 and Tnfa) in those DN mice (Puthanveetil et al., 2015). Similarly, the upregulation of lncRNA GM6135 expression in DN mice also promoted inflammation by increasing the expression of *TLR4*, which is a component of TLR‐mediated signaling that is responsible for the secretion of pro-inflammatory cytokines (Ji et al., 2019).

In contrast, Myocardial infarction-associated transcript (MIAT) lncRNA is a protective lncRNA for the renal oxidative stress, by increasing the *Nrf2* expression, a regulator of the antioxidant genes in DN mice (Zhou et al., 2015). In an animal model of DN, MIAT expression negatively correlated with serum creatinine level (Zhou et al., 2015), indicating that higher lncRNA MIAT expression is important for a healthy kidney. In contrast, another study showed that silencing the MIAT expression prevented myofibroblast formation in kidney fibrosis (Bijkerk et al., 2019), thus implying that MIAT is a pro-fibrotic lncRNA in tubulointerstitial fibrosis. Interestingly, in this same study (Bijkerk et al., 2019), it was the lncRNA RIAN that exhibited the anti-fibrotic role in tubulointerstitial fibrosis. Different roles of MIAT in mediating kidney injury or fibrosis can partially be explained *via* its interaction with miRNA targets in different pathways and mechanisms. For example, MIAT increased aortic vascular smooth muscle cell survival by inhibiting the expression of miR-145 (Chen et al., 2019), whereas MIAT promoted cardiac fibrosis *via* suppressing the miR-24 expression, a regulator of cardiac fibrosis genes (Eissa et al., 2016b). Therefore, it is important to unravel these molecular interactions to understand the exact mechanisms of DN progression.

#### LncRNA-MiRNA Interactions in Oxidative Stress and Inflammation

Several lncRNAs can regulate oxidative stress and inflammation in DN. Anti-oxidative stress lncRNA, LINC01619 expression was downregulated in DN patients’ renal tissues, and since LINC01619 acts a sponge to miR-27a, and this reduction of LINC01619 expression causing miR-27a-mediated inhibition of *FOXO1* expression, thus consequently leads to the ER stress and podocyte cells injury in DN (Bai et al., 2018). Intriguingly, miR-27a also negatively regulates *NRF2* and *PPARG* expressions, which are the anti-inflammatory factors (Hou et al., 2016; Song et al., 2018; Zhou et al., 2018). Thus, restoration of LINC01619 expression can prevent oxidative stress, inflammation, and podocyte cell injury in DN. Unlike LINC01619, the pro-inflammatory lncRNA, MALAT-1 was increased in DN rats and was shown to inhibit miR-23c expression by competitive binding, and thus increasing the expression of miR-23c targets, the pro-inflammatory proteins (ELAVL1, NLRP3, CASP1, and IL1B) in human kidney proximal tubular epithelial cells (Li et al., 2017b). Another miRNA target of MALAT-1 is miR-449, in which the suppression of miR-449 action led to an increase of the *PAI1* expression (Fawzy et al., 2018). This suppression of miR-449 expression correlated with the reduced GFR readings and increased uric acid and triglycerides in the blood of DN patients (Fawzy et al., 2018). Similar to MALAT-1, pro-inflammatory lncRNA, GM6135 increases the expression of *Tlr4*, by competitive binding to miR-203-3p, thus further activates the secretion of pro-inflammatory cytokines in DN mice (Ji et al., 2019). Interestingly, miR-874 also negatively regulates the *Tlr4* gene expression (Yao et al., 2018). Thus further works are needed to determine whether lncRNA GM6135 could interact with miR-874 and elevates *TLR4* expression. Nevertheless, inhibition of these pro-inflammatory lncRNAs may alleviate the inflammatory response in renal cells *via* the up-regulation of their target miRNAs.

### Autophagy and Apoptosis

#### MicroRNAs in Autophagy and Apoptosis

Prolonged oxidative stress and inflammation can induce autophagy, apoptosis, and fibrosis in the kidney. In a study of human renal proximal tubular cells treated with high glucose concentration, overexpression of miR-155-5p led to the suppression of Sirtuin-1 (*SIRT1*) expression, which is a well-known anti-apoptotic, anti-fibrotic and anti-inflammatory factor, and together with reduction of the two autophagy genes, *LC3-II* and *ATG5* (Wang et al., 2018e). Consistent with this, in microalbuminuria patients, urinary exosome expression of miR-130a and miR-145 were higher, whereas miR-155 and miR-424 expression were reduced (Barutta et al., 2013). Another pro-fibrotic miRNA is the miR-133b, and its low expression in DN patients was in response to *TGFB1* induction (Eissa et al., 2016b). This increased miR-133b expression inhibits E-cadherin (*CDH1*) and *SIRT1* expression, as shown in DN mice (Sun et al., 2018a). Inhibition of miR-133b and miR-199b expression attenuated TGFB1 action, *via* the upregulation of *SIRT1* expression (Sun et al., 2018a). Similar to *SIRT1*, Sirtuin-7 (*SIRT7*) expression is also important to prevent podocyte cells apoptosis, and low *SIRT7* expression in hyperglycemic podocyte cells and DN mice, was due to being negatively regulated by miR-20b (Wang et al., 2017) Besides that, the miR-30 family members are also anti-fibrotic miRNAs, in which their expressions are reduced in DN patients (Eissa et al., 2016b). Their common target, a connective tissue growth factor (*CTGF*), is a potent regulator for fibrosis, and its expression was higher in DN (Wu et al., 2014). Another regulator of *CTFG* is miR-26a, and its expression was low in DN patients and animal models (Koga et al., 2015).

In terms of apoptosis and cell death, miR-503 expression was higher in DN mice, and it negatively inhibits the expression of E2F transcription factor 3 (*E2f3*), and E2f3 is the transcription factor that is involved in the regulation of cell apoptosis, differentiation, and development (Zha et al., 2019). Reduction of the *E2f3* expression leads to podocyte cells apoptosis and injury, induced by high glucose (Zha et al., 2019). Importantly, in the mice model of DN treated with Losartan (a drug for kidney disease treatment), the prevention of DN progression in those mice was evident with the inhibition of miR-503 action (Zhu et al., 2016), therefore confirming the role of miR-503 in contributing to podocyte cell death. Similarly, miR-181b is also the pro-apoptotic miRNA, and its expression was high in DN patients (Zhu et al., 2019). Increased miR-181b expression caused a reduction of *TIMP3* expression, and inhibition of miR-181b caused up-regulation of *TIMP3* expression together with increased mesangial cell viability (Zhu et al., 2019). Other pro-apoptotic miRNAs are the miR-770-5p that negatively regulates TP53-regulated inhibitor of apoptosis-1 (*Triap1*) expression in mice podocytes (Zhang et al., 2019); miR-320 that negatively regulates the transcription factor, *Mafb* in DN mice (He et al., 2019); miR-182-5p that negatively regulates *FOXO1*, actin scaffolding protein (*CD2AP*), and the regulator of cell death, *BCL2* expressions in both human and animal models of DN (Stittrich et al., 2010; Peng et al., 2013; Ming et al., 2019); miR-134-5p that negatively regulates *Bcl2* expression in DN mice (Qian et al., 2018); and all of these miRNAs were upregulated in DN and thus contributing to podocyte cell death. In contrast to that, miR-29a is anti-apoptotic miRNA in which its expression was low in DN mice that negatively regulates cannabinoid type-1 receptor (*Cnr1*) expression (Lin et al., 2014; Tung et al., 2019), and overexpression of miR-29a in animal model of DN prevented the secretion of pro-inflammatory and pro-fibrogenic factors, consequently attenuated renal hypertrophy (Tung et al., 2019). These findings provide the basis of using miRNAs as a therapeutic strategy to prevent or reduce the progression of DN. However, it is important to note that many of these miRNAs also regulate different targets at the same time (Simpson et al., 2016), thus may unnecessarily causing dysregulation in a completely different pathway. Therefore, recent studies have embarked to characterize and develop these miRNA-based therapies for DN.

#### LncRNAs in Autophagy and Apoptosis

In terms of renal autophagy and apoptosis, lncRNA GM5524 expression was increased in DN mice and regulated these processes, upon stimulation with high glucose (Feng et al., 2018). GM5524 inhibits anti-apoptotic *BCL2* expression and activates the LC3/ATG signaling pathway to promote autophagy (Feng et al., 2018). LncRNA GAS5 is also a pro-apoptotic lncRNA, in which GAS5 promoted the human renal tubular epithelial cells apoptosis under high glucose environment (Lv et al., 2019) In contrast to that, lncRNA GM15645 expression was low in DN mice and podocyte cells upon stimulation of high glucose, and this lncRNA acts opposite to lncRNA GM5524 in the regulation of podocyte cells apoptosis and autophagy (Feng et al., 2018). Similar to the anti-apoptotic effects of GM15645, lncRNA Taurine upregulated-1 (TUG-1), was downregulated in the DN and hyperglycemic condition, with evidence of renal damage and loss of podocyte cells in DN patients and animal model (Lei et al., 2018; Shen et al., 2019). Loss of TUG-1 expression reduces peroxisome proliferator-activated receptor gamma coactivator 1-alpha (*Ppargc1a*) expression, as TUG-1 binds to the promoter region of the *Ppargc1a* gene to increase its expression (Long et al., 2016). Consequently, loss of TUG-1 expression increases the tumor necrosis factor receptor-associated factor 5 (*Traf5*) expression and stimulates the podocyte cell death (Lei et al., 2018). Restoration of TUG-1 expression inhibits Traf5 protein production, not its mRNAs expression, and ameliorates podocyte cell survival (Lei et al., 2018). Another anti-apoptotic lncRNA is lncRNA NR_033515, in which its circulating expression was higher in DN patients, and this NR_033515 lncRNA can inhibit apoptosis by increasing the expression of proliferation genes (*PCNA* and *CCND1*) and fibrotic genes (*P38, ASK1, FN, and α-SMA*) (Gao et al., 2018). Recently, Cancer susceptibility candidate 2 (CASC2) lncRNA has diagnostic potential for renal failure, as its reduced expression correlated with most significant renal damage in DN patients (Wang et al., 2018a). CASC2 prevents podocyte cell apoptosis through the inhibition of JNK signaling (Yang et al., 2018a). Although these findings of lncRNAs’ role in DN progression is still lacking, there are potential biomarkers and therapeutic targets from these lncRNAs, particularly the anti-inflammatory lncRNA, MIAT, and anti-apoptotic lncRNA, TUG-1.

#### LncRNAs-miRNAs Interactions in Autophagy and Apoptosis

In terms of renal autophagy and apoptosis, lncRNA NR_033515 expression was higher in DN patients, and it inhibits miR-743b-5p action, and consequently increases the proliferation genes (*PCNA* and *CCND1*), and fibrogenesis genes (*P38, ASK1, FN, and α-SMA*) (Gao et al., 2018). Similarly, LncRNA 1700020I14Rik inhibits the miR-34a-5p expression and increases the Sirt1/Hif-1α signaling pathway, thus promotes the cell proliferation and fibrotic gene expressions (*Tgfb1*, *Fn*, and *Col4a1*) in DN mice (Li et al., 2018a). The pro-apoptotic role of lncRNA GAS5 was due by its competitive binding towards miR-27a (Lv et al., 2019). In hyperglycemic condition, GAS5 inhibits the regulatory action of miR-27a towards the BCL2 Interacting Protein 3 (*BNIP3*), thus causing an upregulation of *BNIP3* expression and renal tubular epithelial cell apoptosis (Lv et al., 2019). Another lncRNA is the lncRNA XIST, in which its expression was high in patients with primary membranous nephropathy, and this high expression of lncRNA XIST was due to Ang II treatment that promotes podocyte apoptosis (Jin et al., 2019). In this study, higher expression of lncRNA XIST inhibited the miR-217 action from suppressing the *TLR4* expression; thus, leads to TRL4 activation of inflammatory and apoptosis effects (Jin et al., 2019). Interestingly, lncRNA XIST expression can be detected in the urine samples (Huang et al., 2014) thus suggesting the potential of this lncRNA as a biomarker for DN. From this evidence of lncRNA-miRNA interactions, there are two types of lncRNA regulations involved. One is that lncRNAs host the miRNAs and positively regulates these miRNAs. The second action is that lncRNAs act as a sponge to inhibit the miRNAs suppression of their target genes. Therefore, by identifying these interactions in the molecular regulation of DN progression, some of these lncRNAs can be used as potential biomarkers and therapeutic targets.

#### CircRNAs-lncRNAs-miRNAs Interactions in DN

Limited evidence is available to elucidate the interaction of circular RNA in DN progression. Only one study in DN mice showed that circRNA_15698 expression was higher in the DN mice, and knockdown expression of circRNA_15698 in mesangial cells led to reduced expression fibrotic genes (*Col1a1*, *Col4a1,* and *Fn*) (Hu et al., 2019). In this study (Hu et al., 2019), circRNA_15698 acts as a sponge to miR-185, which in returns upregulates *TGFB1* expression and promotes the ECM protein production. Therefore, inhibition of circRNA_15698 expression can prevent *Tgfb1*-mediated ECM accumulation and fibrosis in DN. Despite that there is limited information available for circRNAs in DN, the current findings imply that these circRNAs may be involved in DN progression, and the most exciting part is how their roles as miRNA sponge may interact with known lncRNA-miRNA interaction discussed above.

### LncRNA-miRNA-Based Treatment for DN

MiRNAs can control the expression of genes involved in disease: thus, many miRNA-based therapies potentially can be as the alternative treatment options. In order for the miRNA-based treatment to work, the application of chemically engineered oligonucleotides to mimic (miRNA mimics) or silence the microRNAs (antagomiRs) were developed (Schena et al., 2013). One example of such techniques is the locked nucleic acid (LNA) molecular inhibitor to suppress a particular miRNA expression or action (Schena et al., 2013). Interestingly, LNA-miR-192 (to reduce miR-192 expression), showed some potential as a treatment for DN, as it reduces the downstream miRNAs expression following the TGFB response, and the expression of critical genes in fibrosis progression in mice model (Putta et al., 2012). In agreement with most of the current treatments are available for DN patients, the application of the miRNA-based treatment in DN is mainly focused on to prevent renal fibrosis. Subcutaneous injection of antagomiR-miR-21 prevented renal fibrosis in chronic kidney disease mice by preventing both glomerular and tubular cell damage (Gomez et al., 2016). Another study focused on miR-29 family, in which the restoration of miR-29b expression in DN mice resulted in TGF-β/Smad3 pathway suppression, reduced collagen matrix accumulation and inflammation (Chen et al., 2014a), thus implying that miRNA-based treatment could potentially be an alternative option for DN.

Although miRNA-based treatment is positively encouraging, the problem lies within the delivery method to exert this miRNA-based treatment. Most miRNAs are known to regulate many other target genes at the same time (Chen et al., 2014b; Li and Rana, 2014); thus, this treatment option may affect other un-related pathways, if they are delivered as a whole-body effect or *in vivo*. Therefore, current research in miRNA-based therapies is shifted to focus on the delivery efficacy and safety, either by using a vector (virus particles or lipid particles) to target a specific route and localization to ensure a local tissue or region effect (Lanford et al., 2009; Simpson et al., 2016). Moreover, consideration of the therapeutic agent’s size is also needed to make sure that it is small enough to cross the endothelium to the organ or site of interest and not will be filtered out by the kidney (Schena et al., 2013). However, for treating DN, this filtration problem could be an advantage to the miRNA-based treatment, as the tubular epithelial cells tend to reabsorb the molecules from the ultra-filtrate, thus reducing the loss (Schena et al., 2013). Therefore, there is some promise that miRNA-based treatments can be used for DN patients, though more future works are needed to validate these, especially in human clinical trials.

Several miRNA-based treatment or drugs have advanced to human clinical trials, though none was for treating DN. One such example is Miravirsen, which is a miR-122 inhibitor (LNA-antisense oligonucleotide) that already entered phase II clinical trials to treat HCV infection in patients (Janssen et al., 2013). Subcutaneous Miravirsen injections resulted in dose-dependent reductions in HCV RNA levels without evidence of viral resistance (Janssen et al., 2013). Similarly, RG-101, which is another miR-122 inhibitor for HCV-infected patients, has already completed a phase I trial (van der Ree et al., 2017). Many other miRNA-based treatments are currently in the development pipeline to initiate clinical trials for different human diseases; therefore, the option to use miRNA-based treatment for DN is a new research interest.

Another possible treatment is using the lncRNAs for DN. Targeting the lncRNA expression is favorable when compared to miRNAs, due to its functional role in transcriptional regulation, tissue-specific expression, cell- and disease-specific dysregulation. Because of the lncRNAs are predominantly in the nucleus, modified antisense oligonucleotides (ASOs) are used to target or silence the lncRNA in the nucleus by initiating the RNase H–dependent degradation (Kurreck et al., 2002). Such examples are the 2′-OMe RNA and LNA modifications at both the 5′ and the 3′ end (Kurreck et al., 2002). Though, the challenge is to make sure that the ASO binding is as intended to the lncRNA specific site, as the tertiary and secondary structures of lncRNAs can block such binding (Adams et al., 2017). Hence, to ensure the binding happens, the only solution is to provide or design more modified ASOs to target a single lncRNA, but the cost of each will be expensive. Moreover, the real challenge is the application of using this lncRNA-based treatment *in vivo*, and similar to miRNA-based treatments, the problems lie within their efficient delivery and efficacy. Another problem lies in the heterogeneous nature and un-conserved intron sequence of lncRNAs (Garitano Trojaola et al., 2013). Therefore, for some lncRNAs, a specific sequence of inhibitor needs to be designed specifically for each of them, mainly if the lncRNAs are from the animal models. Hence, further works need to be done to determine the possibility of using these miRNAs/lncRNAs-based treatments in DN progression as a future treatment option.

## Conclusion

Many ncRNAs (miRNAs, lncRNAs, and circRNAs) are actively discovered to be involved in DN progression due to their regulations of the critical genes in these processes. Since most of these ncRNAs are stable in the biological fluids and can be identified easily without the surgical approach, they provide a strong basis as the potential biomarkers or therapeutic targets. Some of these ncRNAs are very specific towards their targets and localization. Although the exact molecular mechanism of DN progression is still unknown, several processes and pathways are known. Among these processes, ncRNAs involve in renal fibrosis and hypertrophy, ECM protein accumulation, cell autophagy, and apoptosis. Thus, concurrent with ncRNAs characterization, some research has proceeded to synthesize and producing ncRNAs-based treatments, with a few of these ncRNAs are already in the clinical trial phase. Therefore, these ncRNAs-based treatments will be an option for the treatment of DN in the future.

## Author Contributions

TL: wrote the manuscript. SSu: drafted and wrote the manuscript. NA and NAA: edited and critically revised the manuscript for intellectual contents. SSh, AA, and RJ: critically revised the manuscript for intellectual contents.

## Funding

The manuscript was supported by the UKM University grant (GUP-2017-020).

## Conflict of Interest

The authors declare that the research was conducted in the absence of any commercial or financial relationships that could be construed as a potential conflict of interest.
